# Comparative Gene Expression Profiling of *P. falciparum* Malaria Parasites Exposed to Three Different Histone Deacetylase Inhibitors

**DOI:** 10.1371/journal.pone.0031847

**Published:** 2012-02-27

**Authors:** Katherine T. Andrews, Archna P. Gupta, Thanh N. Tran, David P. Fairlie, Geoffrey N. Gobert, Zbynek Bozdech

**Affiliations:** 1 Eskitis Institute for Cell and Molecular Therapies, Griffith University, Queensland, Australia; 2 Queensland Institute of Medical Research, Queensland, Australia; 3 School of Biological Sciences, Nanyang Technological University, Singapore, Singapore; 4 Institute for Molecular Bioscience, The University of Queensland, Brisbane, Queensland, Australia; Université Pierre et Marie Curie, France

## Abstract

Histone deacetylase (HDAC) inhibitors are being intensively pursued as potential new drugs for a range of diseases, including malaria. HDAC inhibitors are also important tools for the study of epigenetic mechanisms, transcriptional control, and other important cellular processes. In this study the effects of three structurally related antimalarial HDAC inhibitors on *P. falciparum* malaria parasite gene expression were compared. The three hydroxamate-based compounds, trichostatin A (TSA), suberoylanilide hydroxamic acid (SAHA; Vorinostat®) and a 2-aminosuberic acid derivative (2-ASA-9), all caused profound transcriptional effects, with ∼2–21% of genes having >2-fold altered expression following 2 h exposure to the compounds. Only two genes, alpha tubulin II and a hydrolase, were up-regulated by all three compounds after 2 h exposure in all biological replicates examined. The transcriptional changes observed after 2 h exposure to HDAC inhibitors were found to be largely transitory, with only 1–5% of genes being regulated after removing the compounds and culturing for a further 2 h. Despite some structural similarity, the three inhibitors caused quite diverse transcriptional effects, possibly reflecting subtle differences in mode of action or cellular distribution. This dataset represents an important contribution to our understanding of how HDAC inhibitors act on malaria parasites and identifies alpha tubulin II as a potential transcriptional marker of HDAC inhibition in malaria parasites that may be able to be exploited for future development of HDAC inhibitors as new antimalarial agents.

## Introduction

Transcriptional control in malaria parasites is relatively poorly understood, however there is increasing evidence that targeting DNA replication/transcriptional regulation represents a potential new therapeutic approach for malaria [1], [2]. Enzymes involved in gene expression and regulation in *Plasmodium falciparum*, the most lethal of the malaria parasites that infect humans, are now beginning to be characterised and their biological roles defined. Histone modification enzymes, including *P. falciparum* histone deacetylases (PfHDACs), are recognised new drug targets for malaria [1], [3]. PfHDACs, together with histone acetyltransferases (PfHATs), reversibly modify the ε-amino groups of lysine residues on the N-terminal regions of histones, thereby contributing to regulation of chromatin-structure dynamics. To date, five putative HDAC-encoding genes have been identified in the *P. falciparum* genome. Two are homologues of the human *S*ilent *I*nformation *R*egulator 2 (Sir2)-related prote*IN* (sirtuin) family (class III HDACs). Although the PfSir2 proteins have been shown to be involved in regulating transcription of some *P. falciparum* virulence proteins, neither of these class III HDACs is essential for parasite survival *in vitro* [4], [5], [6]. Of the other three PfHDACs, little is known about their biological function, however the class I HDAC homologue (PfHDAC1) has recently been shown to be a target of antimalarial HDAC inhibitors [7].

**Figure 1:**
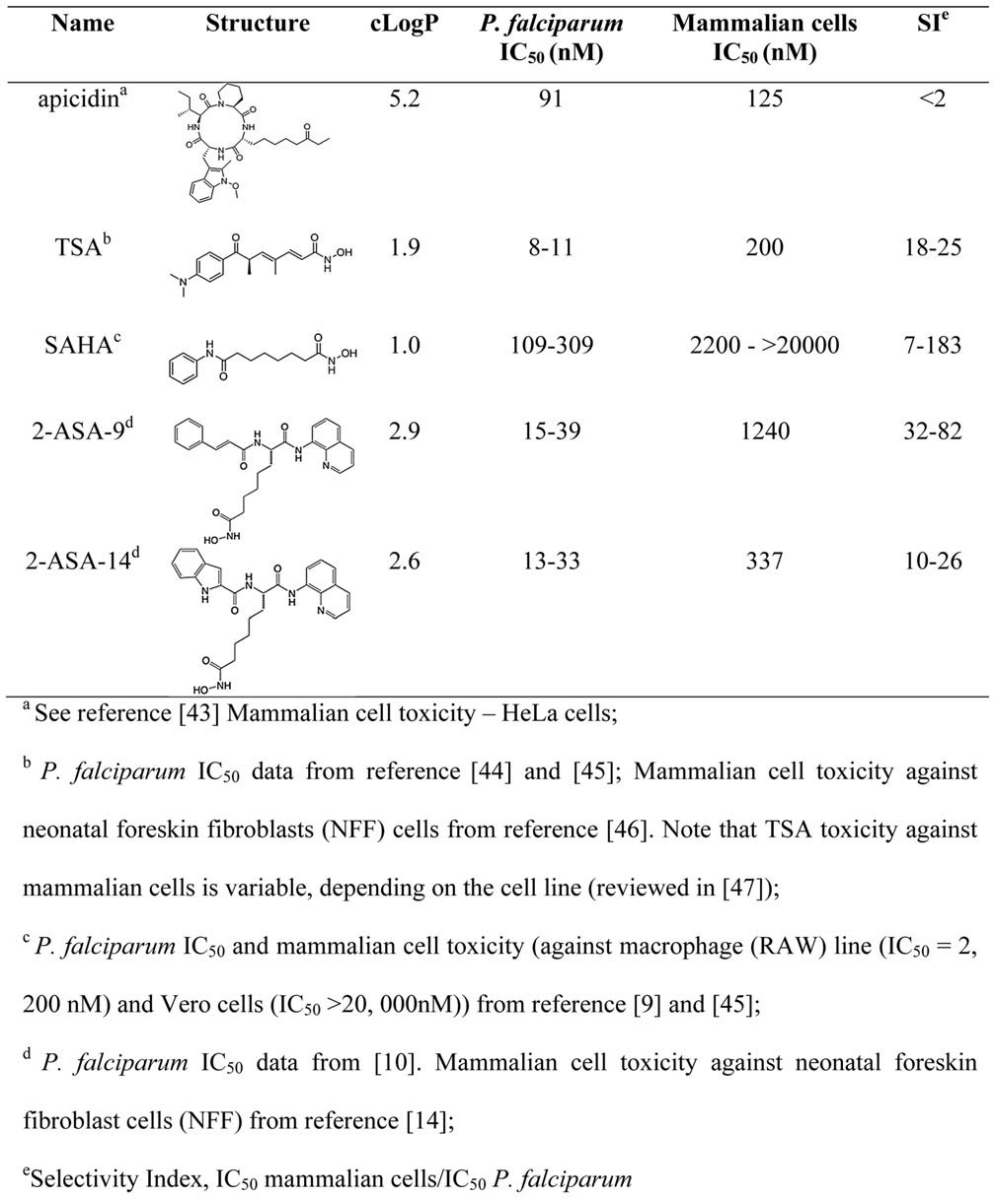
*In vitro* profile of different HDAC inhibitors.

Pan-inhibitors of both class I and II HDACs, such as the cyclic tetrapeptide apicidin and the hydroxamate trichostatin A (TSA), have potent antimalarial activity *in vitro* (Figure 1) [8]. These compounds cause hyperacetylation of *P. falciparum* histones, indicating inhibition of one or more PfHDACs [8]. Unfortunately, both apicidin and TSA suffer from metabolic instability and neither is parasite-selective (Figure 1), so without modifications that overcome these problems, both are unsuitable antimalarial drugs. To address these issues, second generation hydroxamate-based compounds are now being pursued, some of which have similar *in vitro* potency against *P. falciparum* as TSA (IC_50_<50 nM) but, importantly, have improved *in vitro* selectivity in killing parasites over host cells (Figure 1) [9], [10]. Like TSA, these compounds are known inhibitors of HDACs, cause hyperacetylation of *P. falciparum* histones, and inhibit deacetylase activity in *P. falciparum* nuclear extracts [9], [10]. Despite this indication of mode of action in the parasite, little is known about subsequent effects of such hydroxamate-based antimalarial compounds on *P. falciparum* gene expression. Such information may be important not only to help understand transcription in the parasite, but also for identifying molecular markers to aid in the development of drugs to specifically target transcription in *P. falciparum*.

To begin to address this, we recently carried out a genome wide gene expression survey to examine the effect of 20 antimalarial compounds, including apicidin and TSA, on *P. falciparum* intra-erythrocytic developmental stages, the life cycle stage that is responsible for the clinical symptoms associated with malaria [11]. TSA and apicidin caused a general deregulation of the intra-erythrocytic developmental cycle transcriptional cascade (∼30–60% of the genome). This dramatic effect was not seen with other antimalarial compounds, including protease inhibitors, kinase inhibitors, and the antimalarial drug chloroquine. These findings of large scale transcriptional alterations by HDAC inhibitors are not surprising, and mirror affects seen with these compounds in higher eukaryotic cells [12], [13]. However, as discussed above, both apicidin and TSA have poor parasite-specific selectivity (Figure 1; SI<30), raising the question as to whether they act against parasites in the same way as more parasite-selective antimalarial HDAC inhibitors. In this study, we investigated this by examining the genome wide transcriptional effects of TSA and two other hydroxamate-based compounds, suberylanilide hydroxamic acid (SAHA; Vorinostat®) and an analogue of 2-aminosuberic acid (2-ASA-9) [14] in *P. falciparum* parasites. Both compounds have potent *in vitro* activity against *P. falciparum*, but better selectivity than apicidin or TSA at the whole cell level for *P. falciparum* compared to mammalian cells (Figure 1). In comparing the transcriptional affects of these compounds, we identified alpha tubulin II as a gene that is commonly up-regulated by all three hydroxamate HDAC inhibitors, but not other antimalarial compounds. We propose that this gene represents a signature of HDAC inhibition in *P. falciparum* that will assist in the development of this class of compounds as antimalarials. Furthermore, we examined whether transcriptional changes caused by the three HDAC inhibitors result in long-lasting or transitory effects, so as to better understand temporal gene regulation in the parasite.

## Results

### Genome wide effect of three hydroxamate-based HDAC inhibitors on *P. falciparum* transcription

**Figure 2:**
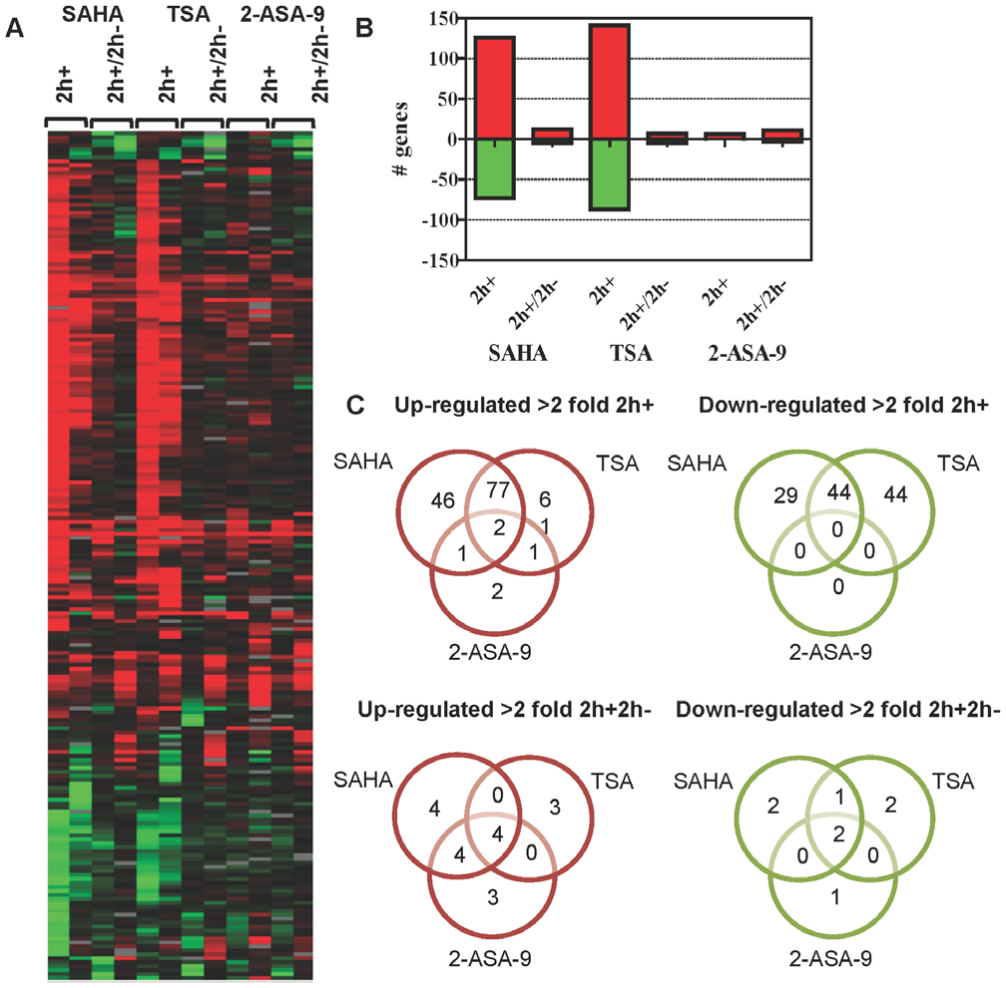
Global transcriptional response of *P. falciparum* to hydroxamate-based HDAC inhibitors. Synchronous 3D7 trophozoite-stage parasites were treated with DMSO (0.05%) or IC_90_ amounts of SAHA, TSA, or 2-ASA-9. RNA was collected from parasites after treatment for 2 h (2 h+), or after 2 h, washing, and culture for a further 2 h (2 h+/2 h−). cDNA was synthesized from RNA and labelled with Cy5 and hybridized against Cy3 labelled 3D7 reference pool. Microarray data, including mRNA abundance ratios between each sample and the 3D7 reference pool, was filtered as described in material and methods. Data shown are genes with >2 fold differential expression induced by the compounds. Heat map showing up- (red), down- (green) regulated or relatively unchanged (black) genes, for two independent biological replicates for each compound (**A**). The total number of genes with altered expression is shown graphically (**B**) and genes commonly or uniquely regulated in 2 h+ or 2 h+2 h− parasites are shown in Venn diagrams (**C**). For (**B**) and (**C**) data are the combined replicates for each compound to show genes commonly up- or down-regulated for each compound and treatment time. For example, 127 genes were up-regulated in common between both biological replicates for parasites treated with SAHA for 2 h (**B**).

To examine the effect of different hydroxamate-based HDAC inhibitors on the *P. falciparum* transcriptome, 3D7 trophozoite-stage parasites (∼32–36 h post invasion) were treated with DMSO (vehicle control), SAHA, TSA, or 2-ASA-9 at their IC_90_ concentrations for 2 h (termed 2 h+). Cells were then harvested for preparation of RNA and microarray analysis. To determine if alterations were transitory, parallel cultures were also treated for 2 h and the compounds then removed by washing, followed by culturing of the cells for a further 2 h (termed 2 h+/2 h−). Cells were harvested, RNA prepared and microarray analysis carried out. Two independent biological replicates were preformed and overall, expression data was obtained for 3,942 genes. Using a 2-fold change in expression as a cut-off, parasites exposed to SAHA, TSA, and 2-ASA-9 for 2 h showed modulated transcription levels of 10–21%, 11–17%, and 2–7% of genes, respectively, for two independent biological replicates (Figure 2; File S1). Microarray data of the biological replicates was compared by calculating Pearson Correlation Coefficients for the DMSO-subtracted data. The two 2 h+ biological replicate data sets presented a significant positive correlation of 0.71 and 0.61 (p-value<0.0005), for TSA and SAHA, respectively. Considering the fact that transcriptional changes induced by HDAC inhibitors represent combinations of the direct and indirect effect on histone modifications in the genome [15], the obtained correlation values indicate a good reproducibility between the two biological replicates. Similarly, the 2-ASA-9 2 h+ replicates showed a good reproducibility where in both cases only a handful of genes were consistently up-regulated (Figure 2). For further evaluation of the reproducibility between the biological replicas, we carried out additional microarray assays with the 0 h DMSO control samples (starting cultures of the two biological replicas). First, each 0 h DMSO sample was hybridized against *P. falciparum* 3D7 cDNA pooled from all intraerythrocytic developmental cycle stages in order to confirm the level of synchronicity in these starting cultures. Comparative analysis showed high levels of correlations between these two microarray results (regression coefficient 0.74 and Pearson correlation coefficient 0.86) which confirm the high level of synchronicity of both starting cultures. In addition, the two 0 h DMSO samples were compared directly by comparative microarray hybridizations including a dye swap experiments (2 microarrays for each swap) in order to assess the progression of the developmental stages in the two starting cultures and technical reproducibility of the microarray analyses. Similarly, the average regression coefficient 0.81 (±0.09) and absolute value of the Pearson correlation coefficient 0.9 (±0.05) indicate good biological and technical consistency of all transcriptomic analyses presented in this study. Data analyses below focus on effects that were in common between different pairs of compounds, or in common to all three compounds

### Short term exposure to hydroxamate HDAC inhibitors results in transient regulation of *P. falciparum* gene expression

**Figure 3:**
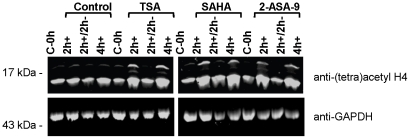
Histone H4 hyperacetylation assay. *P. falciparum* 3D7 infected erythrocytes were cultured *in vitro* with IC_90_ concentrations of TSA, SAHA or 2-ASA-9 for 2 h (2 h+), for 2 h followed by washing and culture for a further 2 h (2 h+/2 h−), or for 4 h (4 h+). Matched control cultures, including a control taken at the start of the experiment (C-0 h) received vehicle only. Parasite protein lysates were prepared and separated via SDS-PAGE, followed by Western blot analysis using anti-(tetra) acetyl histone H4 or anti-GAPDH antisera as a loading control.

To investigate whether transcriptional alterations caused by short-term (non-lethal) exposure to hydroxamate HDAC inhibitors is long lasting or transitory; the effect of the three different HDAC inhibitors was compared after 2 h exposure or after 2 h exposure followed by washing to remove compounds and an additional 2 h culture (2 h+2 h−). First, Western blot with an anti-(tetra) acetyl-H4 antisera was used to examine *in situ* acetylation of *P. falciparum* histone H4. As expected, all three HDAC inhibitors, but not DMSO vehicle, caused hyperacetylation of histone H4 following 2 h or 4 h continual exposure to the compounds. In contrast, the H4 acetylation state returned from a hyperacetylated state in 2 h+ parasites, to a similar level as the controls in 2 h+/2 h− parasites (Figure 3).

Next the genome-wide transcriptional changes in 2 h+ versus 2 h+/2 h− parasites were examined. The total number of genes with altered expression in 2 h+2 h− parasites was ∼1–5% of genes on the array, compared to ∼2–21% in 2 h+ parasites, depending on the compound being examined (2-fold cut off; File S1). Of the genes that were up- or down-regulated after 2 h exposure to SAHA, more than 85% were no longer regulated >2-fold after washing off the compound and culturing for a further 2 h (File S2). For TSA and 2-ASA-9, more than 80% and ∼70% of genes, respectively, that were >2-fold regulated in 2 h+ parasites, were no longer regulated in 2 h+/2 h− parasites (File S2).

### Hydroxamate HDAC inhibitors regulate a small common gene set in *P. falciparum*

**Figure 4:**
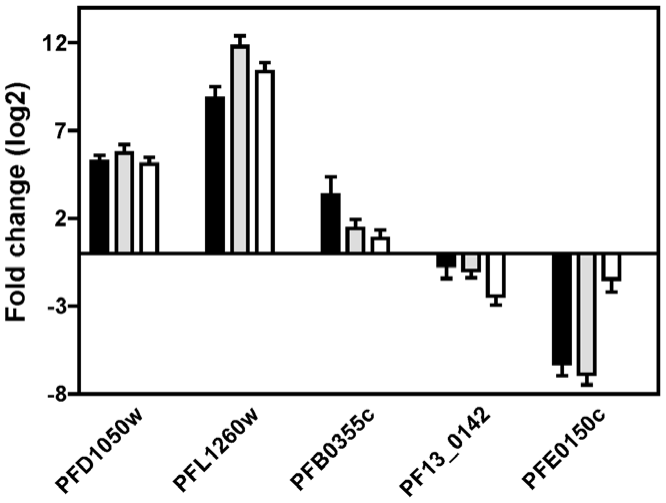
Validation of up- and down-regulated gene expression by quantitative PCR. Quantitative RT-PCR was carried out on five genes using RNA obtained from cells treated with DMSO or compound for 2 h (2 h+). PFD1050w (alpha tubulin II) and PFL1260w (putative hydrolase/phosphatase) were up-regulated in both biological replicates for each compound in microarray analyses; PFB0355c (SERA2) was up-regulated in at least one biological replicate for each compound; and PF13_0142 (a putative spliceosome component) and PFE0150c (a putative kinase) were both down regulated at least one replicate for TSA and SAHA, but not regulated by 2-ASA-9. Relative fold change in expression levels were calculated using 2^−ΔΔCt^ method where histone H4 gene was used as internal control.

Only two genes, alpha tubulin II (PFD1050w) and a putative hydrolase/phosphatase (PFL1260w), were commonly up-regulated by TSA, SAHA and 2-ASA-9 in all replicates after 2 h exposure to the compounds (Figure 2C). This was the case using both a 2- and 3-fold cut-off. Alpha tubulin II, but not the hydrolase/phosphatase, was also >2-fold up-regulated by all three compounds in 2 h+/2 h− samples. No genes were commonly down-regulated by the three compounds. The up-regulation of alpha tubulin II and the putative hydrolase/phosphatase was confirmed using quantitative RT-PCR (Figure 4). The expression of three additional genes was also examined by RT-PCR. Serine repeat antigen 2 (SERA-2; PFB0355c), which was up-regulated >2-fold in at least one replicate for all three inhibitors in microarrays experiments, was also up-regulated by all compounds in quantitative RT-PCR, but only >2-fold by SAHA. PF13_0142 (putative U6 snRNA-associated Sm-like protein (LSm6)) and PFE0150c (putative 4-diphosphocytidyl-2c-methyl-D-erythritol kinase (CMK)) were down-regulated approximately >1.5–2-fold in at least one 2 h+ replicate by SAHA, TSA or 2-ASA-9 treatment in microarray experiments. Both genes were also down-regulated by quantitative RT-PCR (Figure 4).

In order to investigate the possibility that alpha tubulin II and the putative hydrolase/phosphatase might represent transcriptional markers of HDAC inhibition in *P. falciparum*, we examined the expression of these genes in other publically available *P. falciparum* microarray data sets. In our previous microarray study showing *P. falciparum* transcriptional changes following exposure to 20 different compounds, alpha tubulin II was up-regulated following exposure to TSA and the cyclic tetrapeptide HDAC inhibitor apicidin [16]. However, in most samples the putative hydrolase/phosphatase was not up-regulated by these two HDAC inhibitors (File S3). Strikingly, with the exception of staurosporine which up-regulated the putative hydrolase/phosphatase in some samples, neither gene was up-regulated in parasites treated with 18 non-HDAC inhibitor compounds (File S3) [11], [15]. Likewise, neither alpha tubulin II nor the putative hydrolase/phosphatase was amongst small sets of genes differentially regulated in 3D7 parasites exposed to chloroquine [17] or the dihydrofolate reductase inhibitor WR99210 [18]. In *P. falciparum* parasites exposed to artesunate [19] or heat stressed [20], alpha tubulin II gene transcription was not regulated compared to controls, however the putative hydrolase/phosphatase transcript was up-regulated. In another study, alpha tubulin II expression was not changed in parasites exposed to the choline inhibitor T4 for 5 h (∼2× or ∼20×IC_50_) or 18 h (∼125×IC_50_). While this gene was up-regulated ∼2-fold in parasites after 24 h exposure with very high (40× IC_50_) amounts of the compound [21], it is difficult to make any direct comparisons of these data with our own given that T4 has been previously shown to act rapidly on both ring and trophozoite stage *P. falciparum* parasites (>85% inhibition following 1 h exposure to ∼20–40× IC_50_ T4) [22]. While the effects of compound kinetics, such as the time taken to reach a given compounds target, need to be considered, our data suggest that alpha tubulin II may be a transcriptional marker of HDAC inhibition in the parasite.

**Figure 5:**
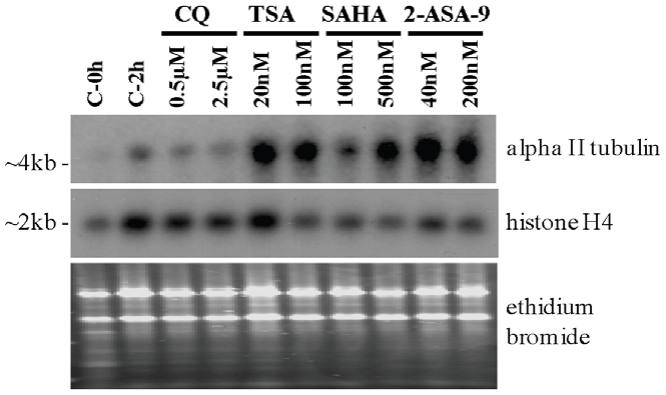
Effect of HDAC inhibitor treatment on transcription of *P. falciparum* alpha tubulin II and histone H4 genes. Synchronous trophozoite-stage K1 *P. falciparum* infected erythrocytes were treated with ∼1×IC_50_ or ∼5×IC_50_ concentrations of chloroquine (0.5 and 2.5 µM), TSA (20 and 100 nM), SAHA (100 and 500 nM), or 2-ASA-9 (40 and 200 nM) for 2 hours. Matched controls taken at 0 h and 2 h (C-0 h and C-2 h, respectively) were treated with 0.05% dimethyl sulfoxide (DMSO). Total RNA was prepared and separated via electrophoresis on an 1.0% agarose gel, followed by transfer and crosslinking to nitrocellulose membrane. The membrane was probed with ^32^P-labeled alpha tubulin II purified PCR product, then stripped and re-probed with ^32^P-labeled histone H4 gel purified PCR product. The histone H4 transcript and ethidium bromide-stained agarose gel is shown to indicate RNA loading levels.

As discussed above, alpha tubulin II was the only gene that was up-regulated by all HDAC inhibitor treatments in our current study and previous studies, but not by short term exposure to other antimalarial compounds. As our microarray and quantitative RT-PCR data was carried out using a chloroquine-sensitive line (3D7), the expression of alpha tubulin II was assessed in a different line to determine if the expression of this gene was also up-regulated in drug resistant parasites. In Northern blot analysis of RNA from *P. falciparum* K1 parasites, an increase in transcript was detected using an alpha tubulin II-specific probe in parasites treated with TSA, SAHA, and 2-ASA-9, but not the antimalarial drug chloroquine (Figure 5).

### Clusters of genes are differentially regulated by different HDAC inhibitors

Even though the three HDAC inhibitors tested in this study are all hydroxamates, there were still a number of differences in the expression changes observed. When data from the two biological replicates for each compound were combined, one cluster of genes had greater than 2-fold increased expression after 2 h treatment with SAHA or TSA but was not up-regulated by 2-ASA-9. Functional pathway analysis of this gene set was carried out against the Gene Ontology (GO) and Malaria Parasite Metabolic Pathways (MPMP) however only one pathway (“*cellular protein metabolic process*”; p = 0.01), had more than 2 genes over-represented (File S4).

Another cluster of genes that showed differential expression in the combined biological replicates are those that were down-regulated more than 2-fold by SAHA and TSA, but not by 2-ASA-9 exposure (Figure 2). Pathways significantly over-represented with more than two genes being present in this gene set include the “*nuclear genes with apicoplast signal sequences*” pathway (p = 0.02) and the “*nuclear genes with mitochondrial signal*” pathway (p = 0.04) (File S4).

Each individual 2 h+ replicate was also examined for functional pathways that were significantly over-represented (p<0.05) using the Malaria Parasite Metabolic Pathway (File S5). In at least one replicate for each compound, merozoite invasion-related protein pathways were found to be significantly over-represented in both up-regulated and down-regulated gene sets. This result is similar to our previous finding in that trophozoite-stage parasites exposed to the non-hydroxamate based HDAC inhibitor apicidin increased transcription of genes belonging to the functional group “*subcellular localization of proteins involved in invasion*” that are normally expressed in schizonts [15]. The “*Sphingomyelin and ceramide metabolism*” pathway was also significantly over-represented in all replicates of the up-regulated gene set (except 2-ASA-9 replicate 1) with four genes being represented: a longevity-assurance (LAG1) domain protein (PFE0405c), a long chain fatty acid elongation enzyme (PFI0980w), dolichol phosphate mannose synthase (PF11_0427), and serine C-palmitoyltransferase (PF14_0155). None of the other pathways were over-represented for more than two replicates for any compound in the up-regulated gene set. Likewise, for the down-regulated gene-sets most of the pathways over-represented were not in common between the compounds and replicates, although as discussed below, there were some similarities for TSA and SAHA.

Gene sets only regulated only in 2 h+2 h− parasites were also analysed for functionally enriched pathways. Few trends, in terms of pathways over-represented for multiple compounds and replicates are apparent although as in 2 h+ parasites (above) merozoite invasion related pathways were represented (File S6).

## Discussion

HDAC inhibitors are being pursued as new drugs for a range of diseases including infectious diseases such as malaria. Despite several studies demonstrating that different HDAC inhibitors have potent *in vitro* activity against Plasmodium parasites, we still know little about how these compounds exert their anti-parasitic affect. The aim of this study was therefore to try to better understand the effects of hydroxamate-based compounds, one of the best studied antimalarial HDAC inhibitor classes [1], on *P. falciparum* transcription. The three inhibitors tested were TSA, SAHA, and 2-ASA-9, a 2-aminosuberic acid-based compound. As shown in Figure 1, all three compounds have potent *in vitro* activity against *P. falciparum* parasites, but different levels of parasite-specific selectivity. Overall, the compounds altered the expression of between ∼2–21% of genes in *P. falciparum* trophozoite-stage parasites after 2 h exposure. This level of transcriptional regulation is similar to that obtained in previous studies using mammalian cell lines where, depending on the HDAC inhibitor and/or cell line used, transcription of 2–22% of genes was altered [12], [13], [23]. The changes caused by the hydroxamate compounds in this study were, however, lower than those we recently observed for a non-hydroxamate HDAC inhibitor, apicidin, which regulated ∼41% of genes after 2 h exposure [15]. It remains to be determined if these overall differences are due to experimental variation, compounds-related effect such as cell permeability, or even differences in molecular target(s) within the parasite.

All three hydroxamates tested in this study caused both up- and down-regulation of genes compared to the controls in at least one replicate (Figure 2). Down-regulation of gene expression might appear to be counter-intuitive given that HDAC inhibitors cause hyperacetylation of histones in the parasite which should lead to transcriptional activation, however similar findings have been previously reported [12], [15]. Down-regulation of gene expression by HDAC inhibitors may be the result of acetylation of histones not normally acetylated at a particular time, therefore blocking access of transcription machinery, or due to transcription of regulatory factors that in turn negatively regulate transcription of other genes [12]. In addition, HDACs are also known to target non-histone proteins in mammalian cells, including transcription factors, cytoskeletal proteins, and nuclear import factors [24]. This may have an indirect effect on transcription of some genes. However, it is not yet known if HDACs target non-histone proteins in Plasmodium parasites and so we can only speculate at this stage on what affects this might have following HDAC inhibitor treatment of the parasite.

An interesting finding resulting from this study was that only two genes were commonly regulated by all three HDAC inhibitors, in all treatments and replicates. The genes are alpha tubulin II, which is normally expressed in asexual stage parasites, sporozoites and male gametocytes stages [25], and a putative hydrolase/phosphatase that is normally expressed in gametocyte and sporozoite stage parasites [26]. The normal transcription levels of these two genes was recently confirmed using RNA-Seq of seven different time points across the asexaul stage life cycle, with alpha tubulin II, but not the putative hydrolase/phosphatise, being expressed [27]. Comparison of our data with other microarray data showed that the expression of the putative hydrolase/phosphatase is altered by treatment with HDAC inhibitors as well as some other antimalarial compounds. In contrast, transcription of alpha tubulin II was not affected by short term treatment with non-HDAC inhibitor compounds including the antimalarial drug chloroquine (Figure 5) [11], [17]. While our finding that such a small set of genes are commonly regulated by HDAC inhibitors in *P. falciparum* may seem surprising, it is similar to a previous study using three different carcinoma cell lines. In that study, just 13 of ∼6,800 genes were regulated more than 2-fold (8 up and 5 down) by TSA, SAHA, and another HDAC inhibitor (MS-275; human HDAC-1 selective) [12]. While we speculate that alpha tubulin II may be a transcriptional signature of HDAC inhibition in *P. falciparum*, additional work is required to confirm this. The compounds used in this study are all known to be pan-HDAC inhibitors as they inhibit the activity of multiple class I and II HDAC isoforms in mammalian cells. Therefore additional studies should include determining if other classes of HDAC inhibitors (e.g, class III HDAC inhibitors) and compounds with different HDAC isoform selectivities also up-regulate alpha tubulin II expression. Likewise, additional studies on the Plasmodium stage-specific effects of HDAC inhibitors on alpha tubulin II expression will be of interest, as will studies on possible effects at the protein level. If alpha tubulin II is confirmed as being a transcriptional marker of HDAC inhibitor action in *P. falciparum*, it may be possible use this, together with other assays such as histone hyperacetylation, as an additional marker for identifying and developing new lysine deacetylase inhibitors for malaria.

In order to better understand the pharmacodynamic action of HDAC inhibitors on malaria parasites, we compared the transcriptional changes occurring following short exposure to the three HDAC inhibitors versus a treatment where parasites were washed and returned to culture. The majority of the changes (∼70% or more) that occurred after 2 h exposure to these compounds were transitory. The concentrations of HDAC inhibitors (IC_90_) and exposure times (2 h) used in this study were selected to be sub-lethal, to try to rule out effects that might be due to cell death. Continual exposure of *P. falciparum* parasites to HDAC inhibitors does kill parasites [10], however stage specific growth inhibition assays carried out as part of this study show a slight, but not significant, effect on growth of trophozoite-stage parasites treated with SAHA or 2-ASA-9 if the compounds are washed off after 2 h and the parasites returned to culture (File S7).

Together, our data indicate that transcriptional control in the malaria parasite is altered by exposure to small molecule HDAC inhibitors that may modify chromatin structure and transcription. It is, however, difficult to confirm whether the different effects are due to differences in physiochemical properties (e.g. ability to permeate cells) or to specific changes due to target-based effects. Despite being related in chemical structure and in their influence on the transcription of two specific genes, overall the three compounds caused very different transcriptional changes. The precise reason for this is unclear. All three compounds cause hyperacetylation of *P. falciparum* histones [8], [9], [10] (Figure 3), supporting their role as inhibitors of *P. falciparum* HDAC enzymes. At the molecular level, all three compounds share a common zinc-binding hydroxamate, and similar linker regions that connect this zinc-binding ligand to the “capping” group, but different components at the other end of the molecule. SAHA and TSA have a single aromatic functional group, while 2-ASA-9 has two aromatic groups in this region (File S8). These changes in chemical composition likely influence the binding of these compounds to target proteins. *In silico* molecular modeling studies in which TSA, SAHA and 2-ASA-9 were docked into a three dimensional homology model structure of PfHDAC1 [10], suggest that the three different capping groups likely all bind in a pocket adjacent to the entrance to the catalytic active site of PfHDAC1. The second capping group of 2-ASA-9 is predicted to occupy a second pocket in the protein surface adjacent to the entrance of the catalytic active site. 2-ASA-9 also differs from TSA and SAHA, in that its amide-NHs can interact via H-bonding with Asp97 at the top of the active site tunnel, which has been suggested to be critical for inhibitor binding [28].

It is also possible that the three compounds differentially inhibit *P. falciparum* HDAC enzymes, which might account for different gene expression profiles in the parasite, although non-specific effects might also be possible. TSA and SAHA, while pan-inhibitors of human HDACs, have recently been found to exhibit different inhibitory profiles against individual human HDAC enzymes [29]. Once all recombinant *P. falciparum* HDAC enzymes become available, it will be important to profile these and other HDAC inhibitors for their specificities on malaria parasite HDACs. There may also be other differences in the action of these compounds against non-histone proteins of the parasite that are yet to be determined. In higher eukaryotic cells, numerous non-histone proteins are now known to be associated with HDACs, including transcription factors, cytoskeletal proteins, and nuclear import factors [24]. Human HDACs are known to deacetylate lysine residues of numerous proteins involved in gene expression [30]. Indeed one proteomic analysis of three human cell lines identified over a thousand nuclear and non-nuclear proteins that were modified by lysine acetylation at thousands of individual sites [31].

Finally, the pharmacokinetic and pharmacodynamic properties of these three HDAC inhibitors have not yet been fully evaluated. However, TSA, SAHA and 2-ASA-9 do have CLogP values spanning 2 log units (1.9, 1.0, and 2.9, respectively; Figure 1) suggesting very different lipophilicities that may translate into different cell membrane permeability, different intracellular distributions of inhibitors in the cytosol and nucleus, and different partitioning into organelles.

In summary, in this study three structurally related HDAC inhibitors (TSA, SAHA, and a 2-aminosuberic acid derivative, 2-ASA-9) have been profiled for their effects on gene expression in *P. falciparum* parasites. Each compound was found to cause genome-wide transcriptional changes, consistent with attenuation of HDAC activity in the parasite. Although impacting on up to 21% of *P. falciparum* genes, many of these transcriptional changes were transitory, highlighting the dynamic nature of transcription in the malaria parasite in response to small molecule inhibitors that target chromatin structure. Despite being related in chemical structure, the three inhibitors had very different overall effects on gene expression profiles in *P. falciparum*. Only a small set of two genes were up-regulated by all three HDAC inhibitors. One of these, alpha tubulin II, may represent the first recognized signature of transcriptional change induced by HDAC inhibitors in *P. falciparum* and could potentially be utilized in functional assays for developing the next generation of improved HDAC inhibitors for malaria. Together these findings provide new insights into the pharmacodynamic effects of a promising new class of antimalarial compounds in *P. falciparum* parasites.

## Materials and Methods

### Compounds

The synthesis, purification and characterization of the 2-aminosuberic acid derivative 2-ASA-9 have been previously described [14]. TSA and SAHA were purchased from Sigma-Aldrich (St. Louis, MO). All compounds were prepared as 10 mM stock solutions in DMSO and stored at −20°C.

### *P. falciparum* culture and treatment

*P. falciparum* parasites were cultured *in vitro* essentially as previously described [10], [32]. Treatment of *P. falciparum* infected erythrocytes with HDAC inhibitors was carried out using synchronous trophozoite-stage parasites (∼32–36 h post invasion) at 5% parasitemia and 5% hematocrit. Two independent biological replicates were prepared for each compound and matched controls. Trophozoite-stage parasites were used in this study as this intraerythrocytic stage is more susceptible to SAHA and 2-ASA-9 than ring stage parasites (File S7). Cultures were treated with compound vehicle (0.05% DMSO), and to ensure that differential effects of the three compounds could be compared, ∼IC_90_ concentrations of compounds were used (SAHA, 200 nM, TSA 50 nM, and 2-ASA-9 80 nM), rather than a standard concentration. The IC_90_ concentrations were determined using an *in vitro* ^3^H-hypoxanthine uptake assay, as previously described [10]. At these concentrations, we have previously shown ∼90% of parasites will be killed in growth assays after 24 h exposure [10]. However, File S7 shows that there was no significant reduction in parasitemia compared to controls 24 h after *P. falciparum* parasites were exposed to SAHA or 2-ASA-9 for only 2 h. In this study, cells were treated for 2 h then harvested (termed 2 h+). In parallel, after 2 h exposure to compound, a second matched replicate was washed gently twice in 10 pellet volumes of pre-warmed parasite culture media and returned to culture for a further 2 h (termed 2 h+/2 h−). Controls that were only exposed to compound vehicle (0.05% DMSO) were harvested at the beginning of the experiment and at the same time points as the test compounds. This percentage of DMSO has been previously shown to have no effect on *P. falciparum* transcription [11]. No morphological changes were apparent in any samples after these exposure times, as determined by microscopic examination (not shown). Prior to isolating RNA, cells were washed once in PBS then stored at −80°C in Trizol® reagent. Samples were processed for microarray analysis as previously described [15], and RNA quality confirmed via agarose gel electrophoresis.

### Microarray analysis

Expression profiling was carried out using a long oligonucleotide platform representing all 5,363 *P. falciparum* genes as previously described [33]. We have previously demonstrated that the technical variability of this microarray platform is within the standards of other microarray technologies reaching a high confidence level (>95%) [11], [34] and that fluorophore-related effects are minimal and do not exceed the general level of confidence of microarray technology [34]. cDNA synthesis and labelling was carried out as described [33] and Cy5 labelled cDNA was hybridized against Cy3 labelled 3D7 reference pool. The data was normalized using Lowess method [35] and processed to filter out spots with at least 95% pixels having signal intensity within 2 standard deviations from background for both Cy5 and Cy3 fluorescence. Each gene profile was represented by an average expression value calculated as an average of all spots representing a particular gene. Hierarchical clustering [36] was carried out in Gene Cluster and visualized using Java Treeview [37]. Processed microarray data are shown in File S9. Microarray data has been submitted and assigned GEO accession number GSE25642. An analysis of correlation between the microarray results of biological replicates was performed using Graphpad Prism Version 5 (Graphpad Software Inc.). First it was determined whether the data were distributed normally, using the “D'Agostino & Pearson omnibus normality test”. This test indicated that the data were not normally distributed (P<0.0001), thus a Spearman correlation (Rho) was employed. All methods used an alpha value of 0.05.

### Hyperacetylation assay

Hyperacetylation assays were carried out as previously described [9], [10] using protein lysates prepared from synchronous trophozoite-stage *P. falciparum* 3D7 parasites cultured *in vitro* with IC_90_ concentrations of TSA, SAHA or 2-ASA-9. Cultures were incubated continually for 2 h (2 h+), for 2 h followed by washing and culture for a further 2 h (2 h+/2 h−), or continually for 4 h (4 h+). Matched control cultures, including a control taken at the start of the experiment (C-0 h) received vehicle only (0.05% DMSO). Infected erythrocytes were lysed with 0.15% saponin (Sigma), parasite pellets washed extensively in phosphate buffered saline pH 7.4 to remove haemoglobin, then pellets resuspended in 1×SDS-PAGE loading buffer. Protein extracts were separated via SDS-PAGE then Western blot analysis carried out. Hyperacetylated histone H4 was detected using anti-(tetra) acetyl histone H4 anti-sera (Upstate, Millipore) and loading controlled using anti-PfGAPDH antisera.

### Quantitative PCR

Microarray data were confirmed using quantitative PCR of selected genes on 7500 Real Time PCR System from Applied Biosystems using the Power SYBR Green PCR Master Mix (Applied Biosystems, Life Technologies; California, USA) according to manufacturer's instructions. C_t_ (threshold cycle) values for each target gene under drug treatment were normalized to the expression level of the respective control condition. Relative fold change in expression levels of these genes under drug treatment was calculated using 2^−ΔΔCt^ method where histone H4 gene was used as internal control. All PCR reactions were carried out in triplicates using samples from biological replicate 1. Primer pairs used were: 5′-atg aga gaa gtc att agt att cat gtt gga-3′ and 5′-aac ttc gtc aac gac ggt ggg ttc taa atc-3′ (PFD1050w); 5′-tgt taa aac aca aat ata acg att cat gtg-3′ and 5′- atc ata acc ttt cat atg aat ata agc acc a-3′ (PFL1260w); 5′-aag agt gaa acc act aca gat gaa tct gc-3′ and 5′ ata ctt ctg cac ctg gtc ttg ctg att cta-3′ (PFB0355c); 5′-gtg gaa agt tta aa agg tcg agc agt aat t-3′ and 5′-ctg tta att cac cat cat aat att ctt cag-3′ (PF13_0142); and 5′-gca tgt gga tat gta cgt tta aat aat gag-3′ and: 5′-gaa tgc att aag gtt gat act tca tta tat g-3′ (PFE0150c).

### Northern blot

Synchronous trophozoite stage K1 *P. falciparum* infected erythrocytes (chlorquine- and pyrimethamine-sensitive) [38] were treated with ∼1×IC_50_ and ∼5×IC_50_ concentrations of chloroquine (K1 IC_50_ ∼350–590 nM [39], [40], [41]), HDAC inhibitors (∼IC_50_s determined based on data in Figure 1), or vehicle alone (0.05% DMSO), for 2 h. Cells were washed once in 10 pellet volumes of cold PBS, lysed in 0.15% saponin for 5 min on ice, centrifuged and the parasite pellet resuspended in 10 volumes of Trizol® reagent. Samples were stored at −80°C prior to isolating RNA and carrying out Northen blot analysis, as previously described [42]. PCR products were generated using gene specific primers to alpha tubulin II (5′-gta cca cgt tgt gtg ttc g-3′ and 5′-tca ttc ata tcc ctc atc ttc tcc-3′) and histone H4 (5′-atg tca gga aga ggt aag g-3′ and 5′-acc tcc aaa acc ata taa agt tct tcc-3′). PCR products were labelled with ^32^P-α-dCTP (Perkin-Elmer, USA) using a Hexa-prime labelling kit (Fermentas, USA) and hybridization carried out at 42°C overnight. Membranes were washed twice at 60°C in 0.5× SSC and 0.1% SDS before autoradiography.

## Supporting Information

File S1 **Number of genes regulated by HDAC inhibitors (>2 fold).**
(PDF)

File S2 **Venn diagrams showing numbers of genes regulated uniquely and commonly in 2 h+ and 2 h+2 h− parasites.** Individual biological replicates for SAHA (A&B), TSA (D&E), and 2-ASA-9 (G&H) are shown. Combined replicate data (box) is shown in C (SAHA), F (TSA), and I (2-ASA-9. Only alpha tubulin II (PFD1050w) was up-regulated in every treatment, treatment time, and replicate. Alpha tubulin II, and two hypothetical proteins (MAL8P1.4 and PF11_0479), were commonly up-regulated in each replicate and treatment time for TSA and SAHA. Alpha tubulin II, c14rRNA.3-5s, and a putative hydrolase/phosphatise (PFL1260w) were commonly up-regulated in each replicate and treatment time for SAHA 2-ASA-9. Alpha tubulin II was the only gene commonly up-regulated for every treatment time and replicate between TSA and 2-ASA-9. No genes were commonly down-regulated in any sample.
(PDF)

File S3 **Gene expression data for alpha tubulin II (PFD1050w) and a putative hydrolase/phosphatase (PFL1260w) in** ***P. falciparum*** **parasites treated at different asexual developmental stages with 20 antimalarial compounds.** Data are extracted from our previously published work [16]. Black bar shows ≥2-fold increased transcript detected compared to matched controls for each time point and replicate tested.
(PDF)

File S4 **Excel spreadsheet showing functional pathway analysis of genes commonly up-regulated or down-regulated in 2 h+ parasites treated with SAHA and TSA, but not 2-ASA-9.**
(XLS)

File S5 **Functional pathway analysis of genes up-regulated (A) or down-regulated (B) in 2 h+ parasites.** The numbers of genes in pathways significantly over-represented (p<0.05) for each biological replicate for parasites treated with SAHA (red bars), TSA (blue bars) and 2-ASA-9 (black bars) is shown.
(PDF)

File S6 **Functional pathway analysis of genes up-regulated (A) or down-regulated (B) only in 2 h+2 h− parasites.** The numbers of genes in pathways significantly over-represented (p<0.05) for each biological replicate for parasites treated with SAHA (red bars), TSA (blue bars) and 2-ASA-9 (black bars) is shown.
(PDF)

File S7 **Stage-specific effect of short exposure of HDAC inhibitors on** ***P. falciparum*** **infected erythrocytes.** Synchronous ring (A and C) and trophozoite (B and D) stage *P. falciparum* infected erythrocytes were treated with 25 nM, 125 nM, or 250 nM SAHA or 2-ASA-9 for 2 h (black bars) or 4 h (white bars) followed by washing and assessing parasite growth 48 h later. Percentage growth (± standard deviation) relative to untreated DMSO controls is shown for 4–5 independent assays. Asterisk indicates a significant difference in % growth compared to untreated control cultures (p<0.05).
(PDF)

File S8 **Schematic diagram of the structures of hydroxamate HDAC inhibitors TSA (A), SAHA (B), and 2-ASA-9 (C) showing the zinc binding group (ZBG), linker region, and capping group(s).**
(PDF)

File S9 **Excel spreadsheet of microarray data.**
(XLS)
