# Supplementary material for: Comparative Gene Expression Profiling of P. falciparum Malaria Parasites Exposed to Three Different Histone Deacetylase Inhibitors
Source: PLoS One. 2012 Feb 27;7(2):e31847. doi: 10.1371/journal.pone.0031847 (PMC3288058; doi:10.1371/journal.pone.0031847)
Supplement: File S1 — Number of genes regulated by HDAC inhibitors (>2 fold). (PDF) [file pone.0031847.s001.pdf]

File S1 Number of genes regulated by HDAC inhibitors (>2 fold)

	Biological Replicate	Up-regulated	Down-regulated	TOTAL	%
**SAHA**					
**2h+**	1	403	418	821	**21**
	2	201	174	375	**10**
	1&2	126	73	199	**5**
**2h+/2h**	1	59	93	152	**4**
	2	121	67	188	**5**
	1&2	12	5	17	**0.4**
**TSA**					
**2h+**	1	388	278	666	**17**
	2	234	195	429	**11**
	1&2	141	87	228	**6**
**2h+/2h-**	1	19	63	82	**2**
	2	131	50	181	**5**
	1&2	7	5	12	**0.3**
**2-ASA-9**					
**2h+**	1	67	11	78	**2**
	2	158	64	222	**7**
	1&2	6	0	6	**0.2**
**2h+/2h-**	1	31	14	45	**1**
	2	114	38	152	**4**
	1&2	11	3	14	**0.3**
