# Supplementary material for: Comparative Gene Expression Profiling of P. falciparum Malaria Parasites Exposed to Three Different Histone Deacetylase Inhibitors
Source: PLoS One. 2012 Feb 27;7(2):e31847. doi: 10.1371/journal.pone.0031847 (PMC3288058; doi:10.1371/journal.pone.0031847)
Supplement: File S2 — Venn diagrams showing numbers of genes regulated uniquely and commonly in 2 h+ and 2 h+2 h− parasites. Individual biological replicates for SAHA (A&B), TSA (D&E), and 2-ASA-9 (G&H) are shown. Combined replicate data (box) is shown in C (SAHA), F (TSA), and I (2-ASA-9. Only alpha tubulin II (PFD1050w) was up-regulated in every treatment, treatment time, and replicate. Alpha tubulin II, and two hypothetical proteins (MAL8P1.4 and PF11_0479), were commonly up-regulated in each replicate and treatment time for TSA and SAHA. Alpha tubulin II, c14rRNA.3-5s, and a putative hydrolase/phosphatise (PFL1260w) were commonly up-regulated in each replicate and treatment time for SAHA 2-ASA-9. Alpha tubulin II was the only gene commonly up-regulated for every treatment time and replicate between TSA and 2-ASA-9. No genes were commonly down-regulated in any sample. (PDF) [file pone.0031847.s002.pdf]

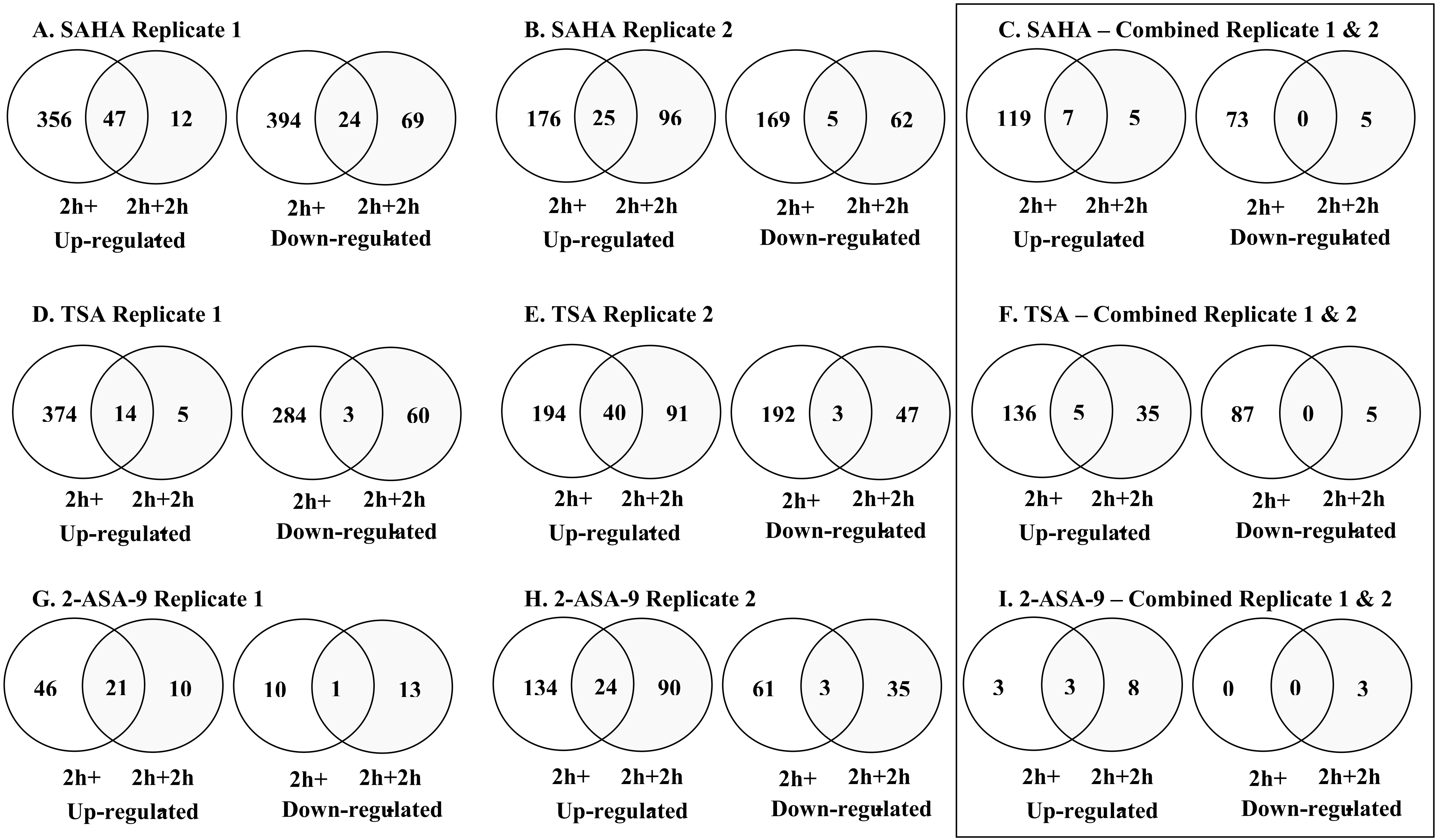

A. SAHA Replicate 1
356
47
12
394
24
69
2h+
2h+2h
Up-regulated
Down-regulated
B. SAHA Replicate 2
176
25
96
169
5
62
2h+
2h+2h
Up-regulated
Down-regulated
C. SAHA – Combined Replicate 1 & 2
119
7
5
73
0
5
2h+
2h+2h
Up-regulated
Down-regulated
D. TSA Replicate 1
374
14
5
284
3
60
2h+
2h+2h
Up-regulated
Down-regulated
E. TSA Replicate 2
194
40
91
192
3
47
2h+
2h+2h
Up-regulated
Down-regulated
F. TSA – Combined Replicate 1 & 2
136
5
35
87
0
5
2h+
2h+2h
Up-regulated
Down-regulated
G. 2-ASA-9 Replicate 1
46
21
10
10
1
13
2h+
2h+2h
Up-regulated
Down-regulated
H. 2-ASA-9 Replicate 2
134
24
90
61
3
35
2h+
2h+2h
Up-regulated
Down-regulated
I. 2-ASA-9 – Combined Replicate 1 & 2
3
3
8
0
0
3
2h+
2h+2h
Up-regulated
Down-regulated
