# Supplementary figures and images for: Comparative Gene Expression Profiling of P. falciparum Malaria Parasites Exposed to Three Different Histone Deacetylase Inhibitors
Source: PLoS One. 2012 Feb 27;7(2):e31847. doi: 10.1371/journal.pone.0031847 (PMC3288058; doi:10.1371/journal.pone.0031847)

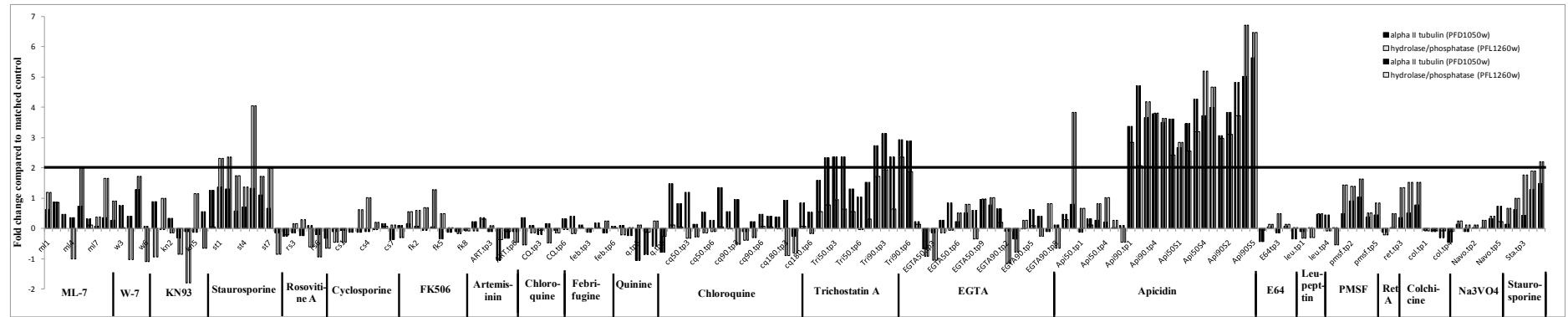

Supplement: File S3 — Gene expression data for alpha tubulin II (PFD1050w) and a putative hydrolase/phosphatase (PFL1260w) in P. falciparum parasites treated at different asexual developmental stages with 20 antimalarial compounds. Data are extracted from our previously published work [16]. Black bar shows ≥2-fold increased transcript detected compared to matched controls for each time point and replicate tested. (PDF) [file pone.0031847.s003.pdf]

A

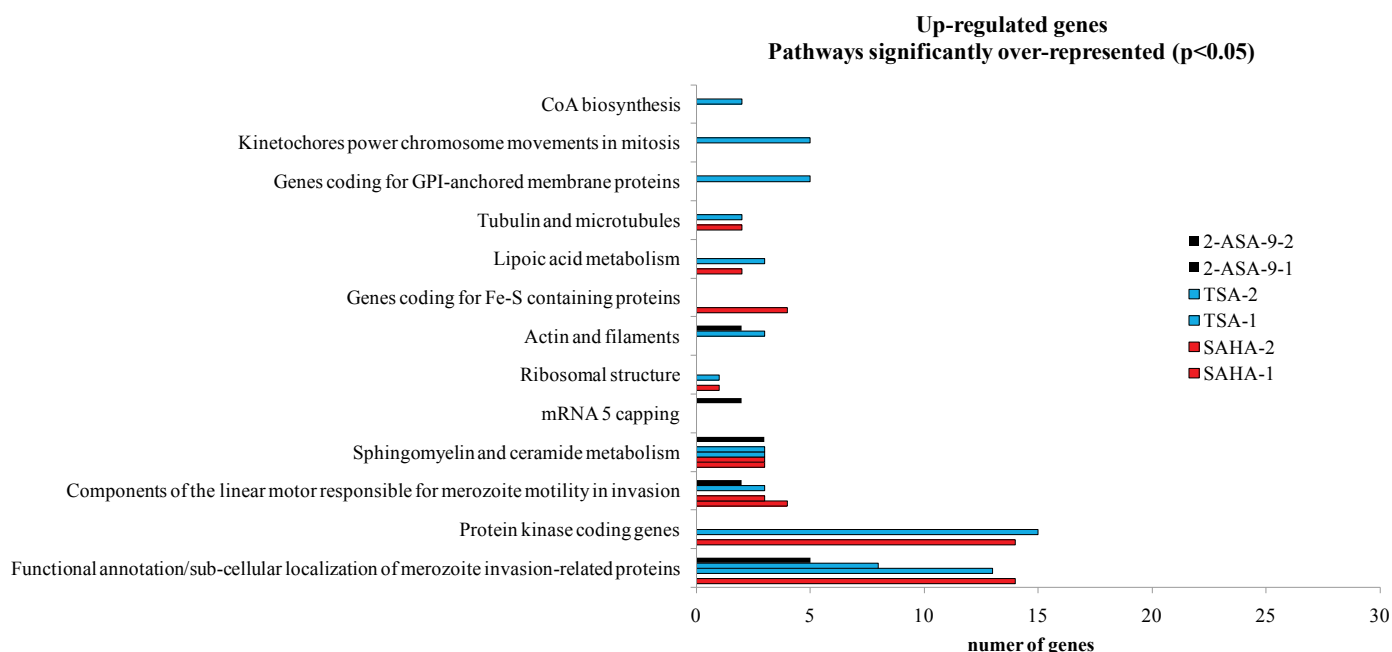

B

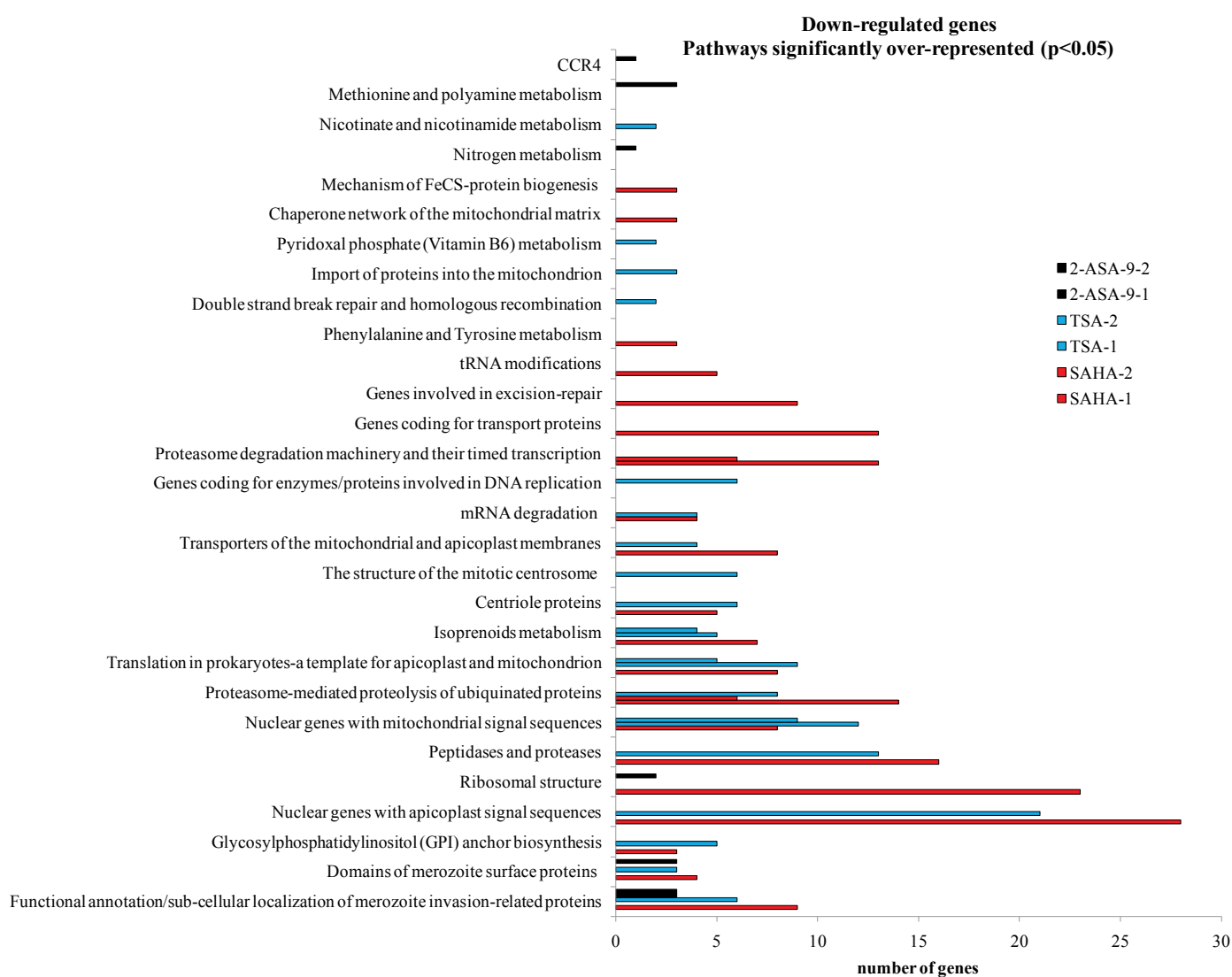

Supplement: File S5 — Functional pathway analysis of genes up-regulated (A) or down-regulated (B) in 2 h+ parasites. The numbers of genes in pathways significantly over-represented (p<0.05) for each biological replicate for parasites treated with SAHA (red bars), TSA (blue bars) and 2-ASA-9 (black bars) is shown. (PDF) [file pone.0031847.s005.pdf]

A.

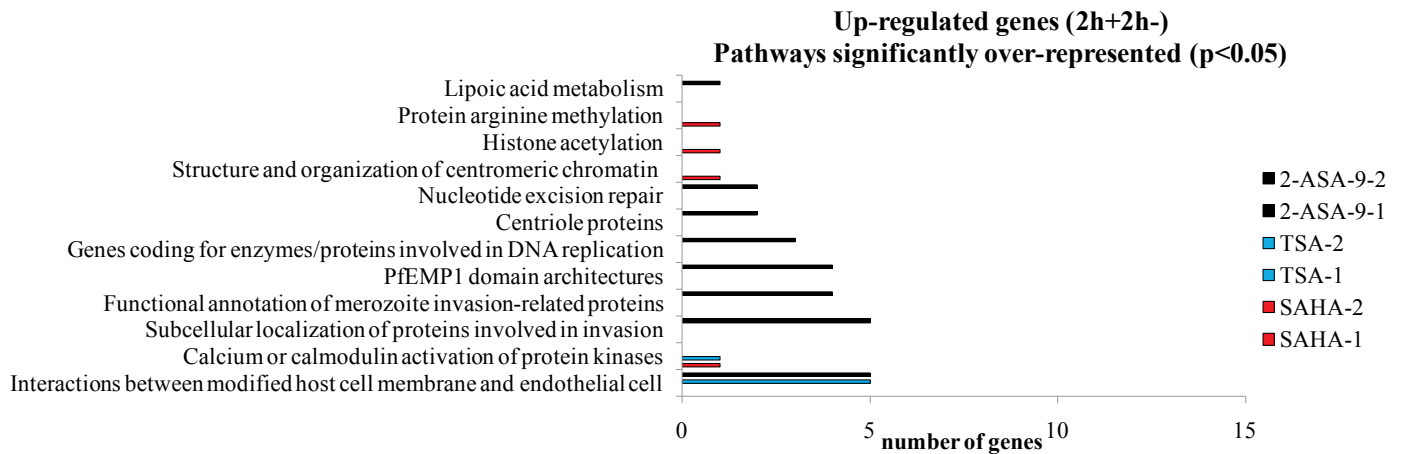

B.

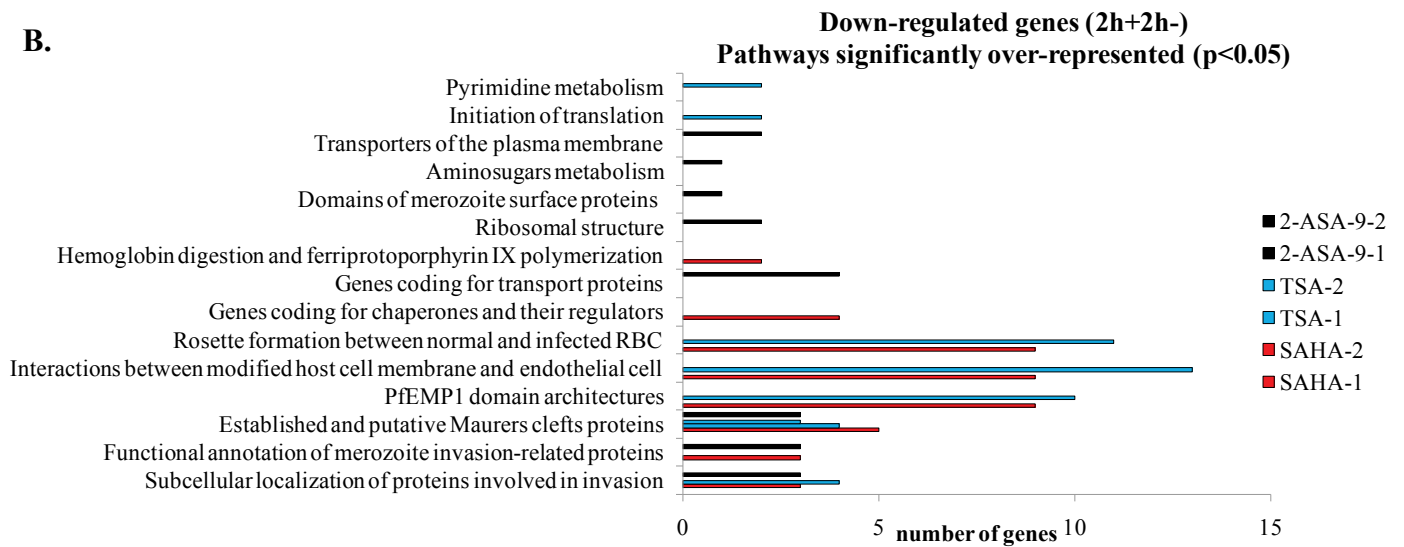

Supplement: File S6 — Functional pathway analysis of genes up-regulated (A) or down-regulated (B) only in 2 h+2 h− parasites. The numbers of genes in pathways significantly over-represented (p<0.05) for each biological replicate for parasites treated with SAHA (red bars), TSA (blue bars) and 2-ASA-9 (black bars) is shown. (PDF) [file pone.0031847.s006.pdf]

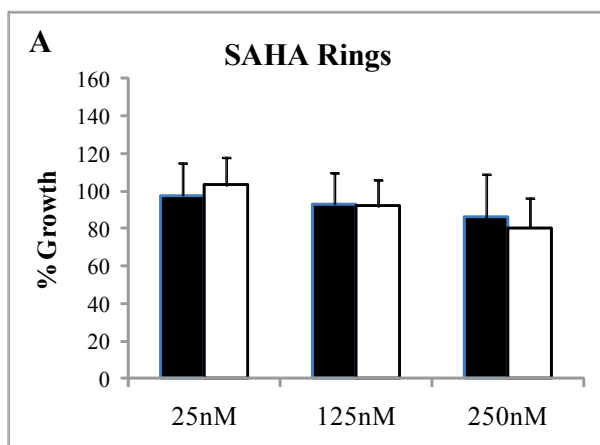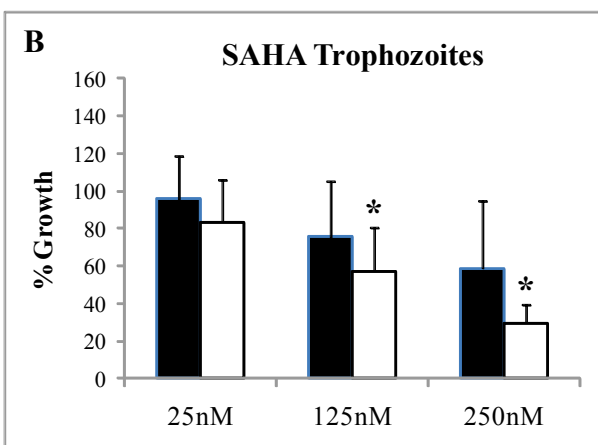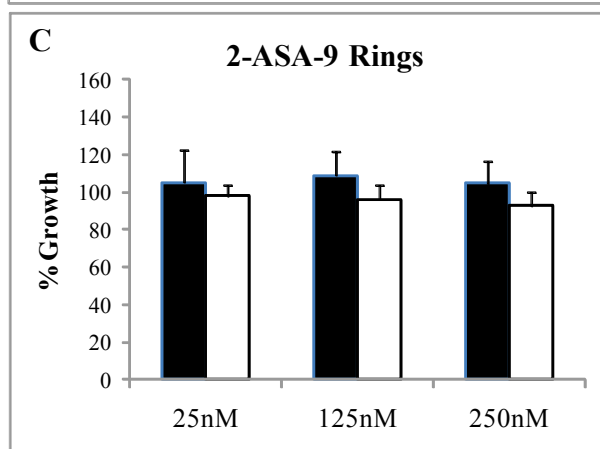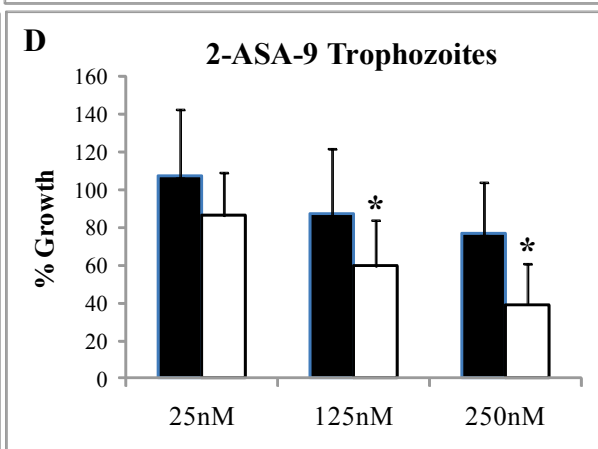

Supplement: File S7 — Stage-specific effect of short exposure of HDAC inhibitors on P. falciparum infected erythrocytes. Synchronous ring (A and C) and trophozoite (B and D) stage P. falciparum infected erythrocytes were treated with 25 nM, 125 nM, or 250 nM SAHA or 2-ASA-9 for 2 h (black bars) or 4 h (white bars) followed by washing and assessing parasite growth 48 h later. Percentage growth (± standard deviation) relative to untreated DMSO controls is shown for 4–5 independent assays. Asterisk indicates a significant difference in % growth compared to untreated control cultures (p<0.05). (PDF) [file pone.0031847.s007.pdf]

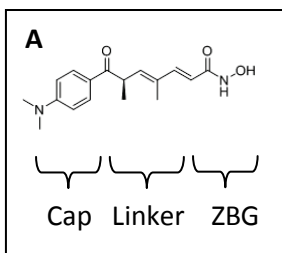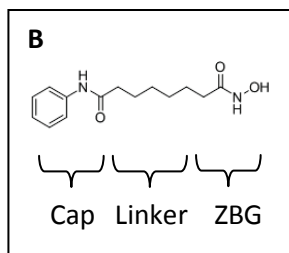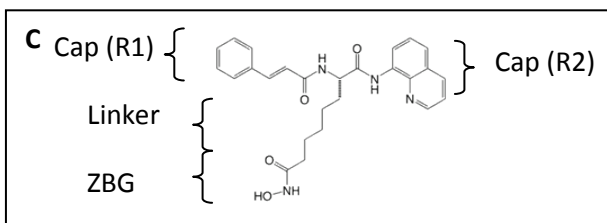

Supplement: File S8 — Schematic diagram of the structures of hydroxamate HDAC inhibitors TSA (A), SAHA (B), and 2-ASA-9 (C) showing the zinc binding group (ZBG), linker region, and capping group(s). (PDF) [file pone.0031847.s008.pdf]
